# Titanium dioxide nanoparticles: a promising candidate for wound healing applications

**DOI:** 10.1093/burnst/tkae069

**Published:** 2025-01-03

**Authors:** Hamed Nosrati, Morteza Heydari

**Affiliations:** Department of Biology, Faculty of Science, Razi University, Kermanshah, Iran; Research Group of Immune Cell Communication, Department of Immune Medicine, Universitätsklinikum Regensburg | UKR, Franz-Josef-Strauss-Allee 11, 93053, Regensburg, Germany

**Keywords:** Titanium dioxide nanoparticles, Wound healing, Tissue engineering, Nanocomposite scaffolds

## Abstract

Effective wound management and treatment are crucial in clinical practice, yet existing strategies often fall short in fully addressing the complexities of skin wound healing. Recent advancements in tissue engineering have introduced innovative approaches, particularly through the use of nanobiomaterials, to enhance the healing process. In this context, titanium dioxide nanoparticles (TiO_2_ NPs) have garnered attention due to their excellent biological properties, including antioxidant, anti-inflammatory, and antimicrobial properties. Furthermore, these nanoparticles can be modified to enhance their therapeutic benefits. Scaffolds and dressings containing TiO_2_ NPs have demonstrated promising outcomes in accelerating wound healing and enhancing tissue regeneration. This review paper covers the wound healing process, the biological properties of TiO_2_ NPs that make them suitable for promoting wound healing, methods for synthesizing TiO_2_ NPs, the use of scaffolds and dressings containing TiO_2_ NPs in wound healing, the application of modified TiO_2_ NPs in wound healing, and the potential toxicity of TiO_2_ NPs.

Highlights

- Titanium dioxide nanoparticles (TiO_2_ NPs) exhibit antioxidant, anti-inflammatory, and antimicrobial properties, making them suitable for wound healing applications.
- Top-down and bottom-up approaches can be used for the synthesis of TiO_2_ NPs.
- TiO_2_ NPs can be modified to enhance their biological properties.
- Scaffolds and dressings incorporating TiO_2_ NPs can effectively support the multifactorial nature of the wound healing process.

## Background

The skin plays a crucial role in protecting against external agents [1]. It serves as a barrier, safeguarding underlying tissues, and is responsible for sensing external stimuli, regulating body temperature, preventing moisture loss, and assisting in the synthesis of vitamin D_3_. To carry out these functions effectively, the structural integrity of the skin is of utmost importance. However, this integrity can be compromised by a range of factors, including injuries, diseases, and surgical procedures [2, 3]. A skin wound refers to damage to the integrity of this tissue, disrupting its normal structure and functions [4]. These wounds can vary in severity, ranging from minor cuts to more significant wounds such as burns, lacerations, and puncture wounds. Severe damage to the skin can pose significant risks to human health and even life, underscoring the importance of the regeneration process in restoring body homeostasis [5, 6].

Wounds can be categorized in various ways based on different factors [7]. They are typically classified into two types: acute and chronic. This classification is primarily based on the healing time and the etiology of the wound [8]. Acute wounds typically result from surgical incisions, mechanical trauma, or burns, and they generally heal faster compared to chronic wounds. In contrast, chronic wounds can take more than three months to heal and are frequently associated with persistent inflammation and significant scarring. Examples of chronic wounds include pressure ulcers, diabetic foot ulcers, arterial ulcers, venous ulcers, wounds related to aging, and chronic wounds resulting from inadequate management of acute wounds [9, 10]. Chronic wounds pose a significant clinical problem, comprising roughly 35% of all wounds and placing a substantial financial burden on both patients and the healthcare system [5, 9].

In recent years, various treatment approaches have been developed for chronic wounds, with skin autografts being considered the preferred and widely accepted method. This approach involves removing healthy skin tissue from unaffected areas of the patient's body and transplanting it onto the wound site [11, 12]. However, this approach has its limitations; it is not appropriate for wounds that cover >60% of the patient's total body surface area. Additionally, there may be insufficient and delayed regeneration of the dermis at the donor site, leading to significant scarring. Complications such as pain, pigmentation disturbance, and hair regeneration issues can also arise [9, 13]. Although skin autografts are currently effective for treating chronic wounds, they may not always be the optimal choice for every patient. Given these limitations, it is crucial to develop alternative approaches and conduct further studies to address the clinical concerns associated with the management and treatment of chronic wounds.

The fields of tissue engineering and regenerative medicine are rapidly evolving, presenting immense potential for effectively addressing the treatment of difficult-to-heal skin wounds [14]. The process of wound healing is intricate, involving the synchronized interaction of diverse cells, proteins, and signaling molecules [15]. While conventional methods have shown effectiveness in treating various types of wounds, tissue engineering offers alternative strategies that can be more effective, particularly for severe or chronic wounds. These innovative approaches involve the utilization of biocompatible materials, cells, bioactive factors, and other biological components to accelerate and enhance the wound healing process [16, 17]. Furthermore, the field of nanotechnology has emerged as a promising area of research in wound healing. Nanomaterials, with their unique characteristics—including nanoscale size, high surface-to-volume ratio, and customizable surface chemistry—exhibit exceptional potential for wound healing applications. Nanomaterial-based approaches have been employed to significantly promote wound healing by supporting cell proliferation and migration, reducing inflammation, increasing the formation of new blood vessels, and enhancing antimicrobial activity against a broad spectrum of microorganisms [18–20].

**Figure 1:**
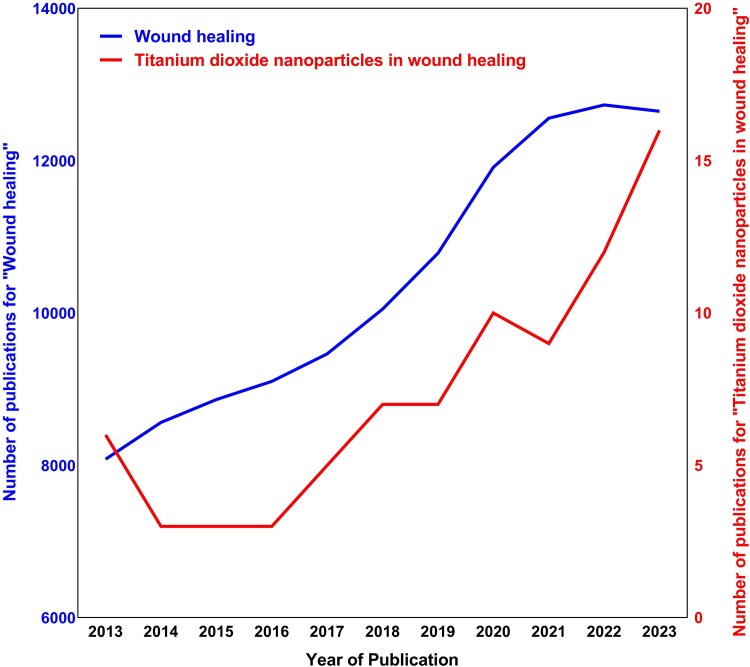
The number of publications on “Wound healing” and “Titanium dioxide nanoparticles in wound healing” per year from 2013 to 2023, according to the PubMed database

In recent years, there has been significant interest in exploring the potential biomedical uses of titanium dioxide nanoparticles (TiO_2_ NPs). Although research specifically targeting TiO_2_ NPs for wound healing is still relatively limited, there has been an increase in exploration in this field over the past decade, according to the PubMed database (Figure 1). TiO_2_ NPs exhibit promising biological properties, including antioxidant, anti-inflammatory, and antimicrobial properties, making them desirable for wound healing [21, 22]. Moreover, TiO_2_ NPs can be modified through processes such as doping, coating, or surface functionalization, which can enhance their biological properties [22, 23]. Additionally, scaffolds and dressings incorporating TiO_2_ NPs have been shown to significantly improve the wound healing process [24–26].

The following sections will discuss the structure of the skin, the wound healing process, the biological properties of TiO_2_ NPs that make them suitable for wound healing applications, methods for synthesizing TiO_2_ NPs, the use of scaffolds and dressings containing TiO_2_ NPs for wound healing, modified TiO_2_ NPs and their role in wound healing, and the potential toxicity of TiO_2_ NPs.

## Review

### Structure of the skin

The skin, the body's largest organ, serves as a protective barrier between internal organs and the external environment [27]. It consists of two main layers: the epidermis and the dermis. The epidermis, the outer layer, is made up of four primary layers: the stratum basale, stratum spinosum, stratum granulosum, and stratum corneum. In the thick skin of the palms and soles, an additional layer known as the stratum lucidum is also present, located between the stratum granulosum and the stratum corneum [5]. The stratum basale is composed of cuboidal to columnar cells, predominantly keratinocytes, which continuously divide and push upwards to replace the cells in the outer layers of the epidermis. As these daughter cells migrate toward the surface, they transition into the outer layers and are eventually shed as dead, keratinized cells [28]. The basal cells also play a critical role in establishing the basement membrane, which is a crucial interface that separates the epidermis from the dermis [29]. Beneath the epidermis lies the dermis, which comprises the bulk of the skin and provides its tensile strength, elasticity, and pliability [30]. This layer contains various cell types, such as fibroblasts, macrophages, and mast cells [31]. However, fibroblasts are the dominant cells and are crucial for the synthesis of extracellular matrix (ECM) components [32]. They also play a critical role in wound healing [33]. The dermis also contains important structures such as hair follicles, sebaceous glands, blood vessels, sweat glands, and nerve endings [34]. Below the dermis is the hypodermis (subcutaneous tissue or superficial fascia), which primarily consists of fat and loose connective tissue. It functions as an energy reservoir, acts as a protective cushion, and insulates the body to prevent heat loss [30, 35]. The subcutaneous tissue is also considered an endocrine organ, converting androstenedione into estrone by aromatase. Additionally, adipocytes produce leptin, a hormone that helps regulate body weight [31, 36].

### The wound healing process

The process of wound healing is the body's effort to restore the disrupted structure and function of the epidermis, dermis, and underlying layers that have been affected by an injury. The healing of skin wounds is a complicated biological process that involves various types of cells and chemical substances [37]. The process of wound healing occurs in four overlapping phases: hemostasis, inflammation, proliferation, and remodeling [38]. The duration of each phase can differ depending on the type and severity of the wound [39].

Hemostasis is the initial stage of wound healing, characterized by the body's natural response to stop bleeding and prevent further blood loss. When an injury occurs, blood vessels in the affected area constrict to reduce blood flow and minimize bleeding. Platelets gather at the site of injury and form a platelet plug. Through a cascade of reactions, these activated platelets trigger the conversion of fibrinogen into fibrin, forming a mesh-like structure that traps blood cells and platelets, resulting in clot formation. This clotting process is reinforced by various clotting factors, ultimately leading to the formation of a stable blood clot [40, 41].

The inflammatory phase begins promptly following an injury and typically persists for ~48 hours. During the inflammatory phase of wound healing, a series of complex processes occur to protect the body against infection and initiate the healing process. The primary goal of this phase is to remove debris or foreign pathogens from the wound site. During this stage, there is a notable influx and migration of immune cells, including neutrophils, lymphocytes, and monocytes [5]. Neutrophils possess multiple mechanisms to eliminate bacteria, foreign particles, and damaged tissue. They are equipped with the ability to engulf and destroy foreign particles through phagocytosis. In addition to phagocytosis, neutrophils can also degranulate and release a range of substances, such as eicosanoids and cationic peptides, as well as proteinases like elastase, proteinase 3, cathepsin G, and urokinase-type plasminogen activator. These substances aid in the removal of bacteria and dead host tissue. As a by-product of neutrophil activity, oxygen-derived free radical species are generated, which possess bactericidal properties [42, 43]. After completing their task, neutrophils may undergo apoptosis, be sloughed from the wound surface, or be phagocytosed by macrophages [42]. Macrophages have multiple functions, including promoting and resolving inflammation, defending the host against pathogens, removing apoptotic cells, and supporting cell proliferation and tissue restoration [44, 45].

The proliferation phase typically occurs 2 to 10 days after the injury, and its progression depends on the interactions between different cell types. During this phase, several important processes take place. Angiogenesis, the formation of new blood vessels, is a key event, providing oxygen and nutrients essential for the healing tissue. This process is crucial for proper wound healing. Additionally, granulation tissue is formed, consisting of new blood vessels, fibroblasts, and ECM components such as collagen, elastin, proteoglycans, and hyaluronic acid. Granulation tissue fills the wound bed and provides a framework for tissue regeneration. Fibroblasts, responsible for producing collagen and other ECM components, proliferate in the wound area. They migrate into the wound bed and deposit new collagen to strengthen the healing tissue. Simultaneously, re-epithelialization occurs as epithelial cells at the wound edges migrate and proliferate, gradually covering the wound surface to restore the skin's protective barrier [37, 46, 47]. Some fibroblasts can differentiate into myofibroblasts, which have contractile properties that aid in wound contraction, reducing the size of the wound and bringing the wound edges closer together. Both fibroblasts and myofibroblasts play a role in synthesizing and depositing collagen, ultimately leading to the gradual strengthening of the healing tissue. Maintaining a delicate equilibrium between the deposition and degradation of ECM components is of utmost importance, as any disruption in this process can lead to abnormalities in scarring [48, 49].

During the remodeling phase, the focus shifts from the formation of new tissue to the remodeling and maturation of the existing tissue. This phase begins 2 to 3 weeks after the injury and can continue for up to 1 year or more. In this phase, apoptosis of unneeded endothelial cells, fibroblasts, and inflammatory cells occurs, helping in scar tissue maturation. Type III collagen is replaced with type I collagen, a process facilitated by matrix metalloproteinase enzymes produced by fibroblasts, macrophages, and endothelial cells. Over time, tensile strength improves as collagen fibers align more finely. However, the reconstructed tissue will never fully regain the strength of undamaged skin [37, 46].

**Figure 2:**
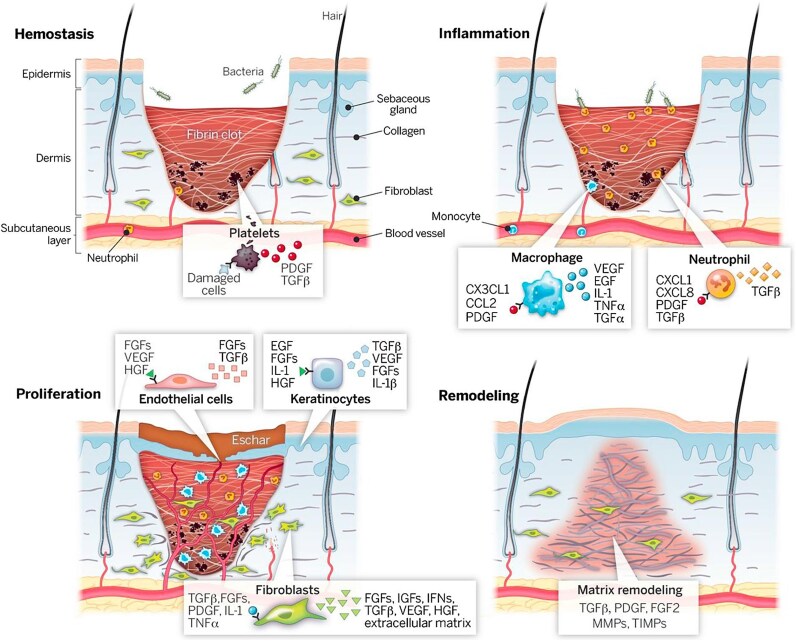
The process of wound healing is typically described as occurring through four primary stages: Hemostasis, inflammation, proliferation, and remodeling. Each phase has distinct molecular and cellular activities taking place. The progression through each stage is orchestrated by signaling molecules secreted by cells involved in the wound healing process. Reprinted with permission from [50]

Figure 2 provides a detailed illustration of the four stages of the wound healing process.

### Biological properties of TiO_2_ NPs for wound healing applications

The promising results obtained from studies on the biological properties of TiO_2_ NPs have established a growing trend of interest in exploring their potential to improve the wound healing process. Excessive production of reactive oxygen species (ROS) can lead to oxidative stress and tissue damage, which can impair the wound healing process [51]. TiO_2_ NPs possess antioxidant properties, protecting the wound from oxidative stress and providing a desirable environment for the progression of the healing process [52]. While inflammation is a natural and necessary part of the initial stages of wound healing, prolonged inflammation can disrupt this process, potentially leading to complications and delaying the formation of new tissue [53, 54]. Studies have demonstrated the anti-inflammatory activity of TiO_2_ NPs [55, 56]. This could support the timely progression of the wound healing process and enhance overall healing outcomes. Additionally, TiO_2_ NPs have antimicrobial properties, effectively inhibiting the growth of a wide range of microorganisms [57]. This characteristic could potentially aid in the prevention of infections, thereby promoting faster and more efficient healing. In this section, some of the most important biological properties of TiO_2_ NPs that make them a promising candidate for the development of advanced wound healing therapies are discussed.

#### Antioxidant and anti-inflammatory properties

ROS are highly reactive molecules containing oxygen atoms [58]. They play a crucial role in various cellular processes and can have both beneficial and detrimental effects. ROS are generated as natural byproducts of aerobic metabolism, particularly in mitochondria [59], but they can also be produced in response to external factors such as pollutants, radiation, or exposure to nanomaterials [60, 61]. Excessive ROS production can lead to oxidative stress, causing damage to lipids, proteins, and DNA, which can disrupt cellular function and contribute to aging, inflammation, and various diseases, including cancer and neurodegenerative disorders [62–64]. Cells neutralize ROS through antioxidants like superoxide dismutase, catalase, and glutathione peroxidase, which help maintain a balance between ROS and antioxidants [65]. Maintaining a balance between ROS and antioxidants is essential for cellular homeostasis. Excessive ROS production can overwhelm the antioxidant defense mechanisms, resulting in oxidative damage and cellular dysfunction [66]. On the other hand, an excessive amount of antioxidants can disrupt normal redox signaling pathways, leading to detrimental effects [67]. Thus, a delicate balance between ROS and antioxidants is crucial for maintaining cellular health and preventing various diseases associated with oxidative stress.

TiO_2_ NPs have been documented to exhibit protective properties against ROS. Akinola et al. evaluated the antioxidant properties of TiO_2_ NPs synthesized using leaf, pod, seed, and seed shell extracts of the kola nut tree (*Cola nitida*) by assessing their antioxidant activity through 2,2-Diphenyl-1-picrylhydrazyl (DPPH) radical-scavenging and hydrogen peroxide scavenging assays. The biosynthesized nanoparticles exhibited antioxidant properties by scavenging DPPH at concentrations ranging from 10 to 80 μg/ml, with responses ranging from 32.61% to 62.06%. Among these nanoparticles, those mediated by kola seed showed the highest antioxidant activity of 62.06% at 80 μg/ml, while TiO_2_ NPs synthesized from the seed shell extract displayed the lowest activity of 32.61% at 10 μg/ml. Furthermore, the biosynthesized TiO_2_ NPs showed significant scavenging activities of 78.45%–99.23% against hydrogen peroxide (H_2_O_2_) in a concentration-dependent manner [68]. In a study conducted by Ajmal et al., TiO_2_ NPs were synthesized using extracts from *Prunus domestica* L. (Plum), *P. persia* L. (Peach), and *Actinidia deliciosa* (Kiwi) peels. The researchers evaluated the antioxidant activity of these nanoparticles through various assays, including the DPPH free radical scavenging assay, hydrogen peroxide scavenging assay, nitric oxide scavenging activity assay, and reducing power assay. The antioxidant activity of the nanoparticles was compared to that of standard compounds like butylated hydroxytoluene and ascorbic acid. The results of the DPPH free radical scavenging assay indicated that the TiO_2_ NPs synthesized using fruit peels exhibited concentration-dependent activity. At lower concentrations, the scavenging activity was relatively low, but it increased as the concentration rose, reaching levels comparable to butylated hydroxytoluene. Plum peel-mediated nanoparticles showed scavenging activity ranging from 10% to 79%, Kiwi peel-mediated nanoparticles ranged from 11% to 69%, and Peach peel-mediated nanoparticles ranged from 8% to 59%, compared to butylated hydroxytoluene, which ranged from 43% to 85%. These measurements were taken at concentrations ranging from 10 to 100 μg/ml Regarding the hydrogen peroxide scavenging assay, the TiO_2_ NPs derived from fruit peels demonstrated good scavenging potential, especially at higher concentrations. At 100 μg/ml, the nanoparticles exhibited scavenging percentages ranging from 57% to 72%. However, at a lower concentration of 10 μg/ml, the scavenging activity was relatively low, ranging from 4% to 9%. Plum peel-mediated TiO_2_ NPs displayed the highest scavenging activity, followed by Kiwi peel-mediated TiO_2_ NPs and Peach peel-mediated TiO_2_ NPs. The nitric oxide scavenging activity assay revealed that the TiO_2_ NPs exhibited concentration-dependent scavenging of NO radicals. At 100 μg/ml, Plum peel-mediated TiO_2_ NPs showed scavenging percentages ranging from 12% to 78%, Kiwi peel-mediated TiO_2_ NPs ranged from 8% to 61%, and Peach peel-mediated TiO_2_ NPs ranged from 4% to 52%. The scavenging activity of the nanoparticles was relatively good compared to ascorbic acid. In the reducing power assay, the fruit peel extract-mediated TiO_2_ NPs displayed good reducing power, particularly at higher concentrations, although it was lower than that of the antioxidant butylated hydroxytoluene. Plum peel-mediated TiO_2_ NPs ranged from 9% to 74% in reducing power, Kiwi peel-mediated TiO_2_ NPs ranged from 7% to 68%, and Peach peel-mediated TiO_2_ NPs ranged from 1% to 59%, whereas butylated hydroxytoluene ranged from 46% to 85% [69]. According to the literature, studies have reported the antioxidant activity of TiO_2_ NPs synthesized using extracts from *Psidium guajava* [70], *Artemisia haussknechtii* leaf [71], and green tea leaf [72].

As previously discussed, excessive and prolonged production of ROS can result in oxidative stress, leading to inflammation and tissue damage. TiO_2_ NPs demonstrate anti-inflammatory characteristics by efficiently scavenging ROS, which helps alleviate oxidative stress and may reduce inflammation and tissue damage. Chahardoli et al. evaluated the anti-inflammatory potential of caffeic acid-mediated synthesized TiO_2_ NPs by assessing their ability to inhibit the denaturation of bovine serum albumin (BSA) protein. The results showed that at concentrations of 31.25 and 500 μg/ml, the synthesized TiO_2_ NPs inhibited BSA denaturation by 70% and 85.7%, respectively. These levels of inhibition were comparable to that of the reference anti-inflammatory drug diclofenac sodium, which inhibited BSA denaturation by 76.7% and 90.9% at the same concentrations [73]. In a study conducted by Bharathy et al., the anti-inflammatory activity of TiO_2_ NPs synthesized using *Gymnema sylvestre* and *Panicum sumatrense* was assessed using the albumin denaturation method *in vitro*. The results demonstrated that the percentage of protein denaturation inhibition increased with increasing concentrations of the synthesized TiO_2_ NPs from 20 to 100 μg/ml. At a concentration of 100 μg/ml, TiO_2_ NPs synthesized from *Gymnema sylvestre* exhibited a 93.52 ± 6.40% inhibitory effect on protein denaturation, while TiO_2_ synthesized from *Panicum sumatrense* showed 82.80 ± 0.37% inhibition. Diclofenac sodium was used as the standard, and showed a 96.25 ± 6.73% inhibitory effect at 100 μg/ml [55]. These findings demonstrate the promising anti-inflammatory activity of TiO_2_ NPs, indicating their potential for applications in biomedical research.

#### Antibacterial activity

TiO_2_ NPs are widely studied for their antimicrobial properties. They exhibit bactericidal photocatalytic activity, possess self-cleaning properties, and are considered safe for use. The antimicrobial activity of TiO_2_ is attributed to the generation of ROS, which affect bacterial cells through various mechanisms, ultimately leading to their death [57, 74]. Bacteria possess enzymatic antioxidant defense systems and natural antioxidants, but when overwhelmed by ROS, a series of redox reactions can occur, altering essential structures and metabolic pathways, ultimately leading to cell death [57, 75]. In this subsection, the specific ways in which cellular structures are affected by TiO_2_ NPs are discussed.

ROS generated by TiO_2_ NPs can cause oxidative damage to various organic structures of microorganisms. One such structure is the cell wall, which serves as the first line of defense against environmental threats. The composition of the cell wall differs depending on the type of microorganism. For example, Gram-positive bacteria have multiple layers of peptidoglycan and teichoic acids while Gram-negative bacteria have a thin layer of peptidoglycan surrounded by a lipid membrane reinforced with lipopolysaccharides and lipoproteins [76, 77]. As a result, the effects of TiO_2_ NPs on the cell wall can vary depending on the type of bacteria. The cell wall of *Escherichia coli* (a Gram-negative bacterium) is sensitive to peroxidation caused by TiO_2_ [78]. Exposure of *Pseudomonas aeruginosa* PAO1 cells to TiO_2_-based nanocomposites has been found to cause the downregulation of certain genes related to the cell wall, including those involved in lipopolysaccharide and peptidoglycan metabolism, pilus biosynthesis, and protein insertion [79].

The cell membrane provides the cell with a flexible cover, permeability, and protection. Many studies on TiO_2_ NPs have focused on the disruption of cell membrane integrity caused by the oxidation of phospholipids by ROS such as hydroxyl radicals and hydrogen peroxide. This oxidation leads to an increase in membrane fluidity, leakage of cellular contents, and ultimately cell lysis [80, 81]. In contrast to the downregulated expression of genes related to the cell wall, genes encoding enzymes involved in lipid metabolism, essential for cell membrane structure, are overexpressed in *P. aeruginosa* PAO1 cells exposed to TiO_2_-based nanocomposites [79]. This suggests that the cells may compensate for initial cell wall damage by reinforcing the cell membrane.

Mitochondria, being a natural source of ROS in aerobic metabolism, produce superoxide anions through the electron transfer process within respiratory chain. However, mitochondria possess enzymatic defense mechanisms, such as superoxide dismutase, glutathione peroxidase, and catalase [57, 82]. The presence of TiO_2_ NPs increases the production of ROS to a level that surpasses the capacity of the enzymatic defense mechanisms to attenuate the damage. This excessive ROS production can lead to a dysregulation in the electron transfer process within the mitochondrial respiratory chain, resulting in further ROS generation [83].

Damage to DNA at the molecular level has a profound impact on the regulation of various microbial processes, such as metabolism, replication, transcription, and cell division [57]. DNA is highly susceptible to oxidative damage, particularly from hydroxyl radicals (OH˙) generated through the Fenton reaction [84]. These radicals may attack the sugar-phosphate backbone or nucleobases of DNA, leading to strand breaks [85]. The detrimental effects of DNA strand modifications are more severe compared to base modifications [57]. Mitochondrial DNA is particularly vulnerable to oxidative damage as it is located in close proximity to cellular sources of ROS [86]. To counteract DNA damage, cells possess enzymatic detoxification systems [57]. Kubacka et al. demonstrated that detoxification and stress-related genes, including those involved in posttranslational modification, protein turnover, chaperones, as well as DNA replication, recombination, modification, and repair, exhibited upregulated expression in *P. aeruginosa* PAO1 cells treated with TiO_2_-UV [79].

Iron is important for cell growth and survival, but malfunction in regulating iron levels can lead to toxicity. Bacteria can regulate iron concentration using siderophores [87, 88]. It has been observed that the expression of genes related to siderophore synthesis and iron transport decreases in the presence of TiO_2_-based nanocomposites, resulting in reduced iron assimilation and cell death [79].

#### Antifungal activity

TiO_2_ NPs have been found to have antifungal activity against various species of fungi. Irshad et al. synthesized TiO_2_ NPs through chemical and green methods, and evaluated antifungal activity of the nanoparticles against *Ustilago tritici*, a pathogenic plant fungus that causes significant damage to wheat crops. The synthesized TiO_2_ NPs exhibited inhibitory effects on fungal growth at various concentrations of 25, 50, and 75 μg/ml [89]. George et al. conducted a study to assess the antifungal activity of zinc oxide nanoparticles (ZnO NPs) and TiO_2_ NPs compared to their bulk-particular forms, as well as Amphotericin-B and Miconazole. They treated eight fungal cultures (*Aspergillus niger*, *Trichophyton, Fonsecaea*, *A. flavus*, *Rhizopus oryzae*, *Fusarium*, *Ramichloridium schulzeri*, and *Cladosporium*) isolated from infected skin and dandruff flakes with the nanoparticles and evaluated their growth inhibition. The results showed that the nanoparticles were more effective than their bulk counterparts and almost equally efficient as Amphotericin-B, with ZnO NPs demonstrating higher antifungal activity than TiO_2_ NPs. The study suggests that both nanoparticles could be potential alternatives to current antifungal drugs in topical applications for treating mycosis, due to their high surface area to volume ratio, non-specific activity, and lower chances of resistance development. Additionally, the use of nanoparticles may reduce the risk of severe side effects associated with traditional antifungal drugs [90]. Other studies have also reported the antifungal activity of TiO_2_ NPs against various fungi, such as *Candida albicans* [91, 92], *Fusarium graminearum* [93], *Penicillium expansum* AL1 [94], *F. oxysporum* [95], *Macrophomina phaseolina* [96], *A. niger*, *A. fumigatus*, *A. nidulans*, and *A. flavus* [97].

As previously mentioned, depending on the type of microorganism, the cell wall has different composition. In fungi and yeast, cell walls are mainly composed of chitin and polysaccharides [57]. Liu et al. focused on evaluating the effects of TiO_2_ NPs on *Pichia pastoris*. They found that the composition of the yeast's cell wall changed in the presence of TiO_2_ NPs, with an increase in chitin content as a response to the ROS effects. The growth inhibition assays revealed that TiO_2_ NPs had an inhibitory effect on yeast cells. The study further investigated the toxic mechanism and demonstrated that the nanoparticles caused toxicity through damage to the cell wall and cell membrane, as well as the accumulation of ROS. Interestingly, the toxicity was not associated with apoptosis or autophagy. The study demonstrated that TiO_2_ NPs possess toxicity towards *P. pastoris*, primarily through impairing the ROS-scavenging system and disrupting the cycle and regulation of reduced glutathione, resulting in ROS accumulation [98]. It has also been demonstrated that, in the presence of TiO_2_ NPs and UV light, hydroxyl radicals, hydrogen peroxide, and superoxide anions initially promote oxidation of the membrane, leading to an unbalance in cell permeability, and potentially causing decomposition of cell walls. This oxidation can inhibit cell respiration by affecting intracellular membranes in mitochondria [57, 99].

### TiO_2_ NPs synthesis methods

**Figure 3:**
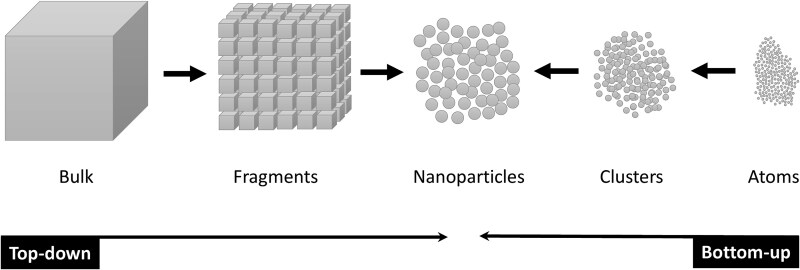
Top-down and bottom-up approaches for nanoparticle synthesis

Nanoparticles can generally be synthesized using either top-down or bottom-up approaches (Figure 3). The top-down approach involves reducing bulk materials into nanoscale particles, typically through physical techniques. In contrast, the bottom-up approach constructs nanoparticles by assembling atoms or molecules into larger nanostructures, often through chemical reactions or self-assembly processes [100, 101].

Each of the physical, chemical, and biological methods of nanoparticle synthesis reflects the underlying principles of either the top-down or bottom-up approach [100]. Physical methods such as ball milling [102], laser ablation [103], and wire explosion process [104], are examples of the top-down approach for synthesizing TiO_2_ NPs. On the other hand, chemical methods, such as the sol–gel method [105], hydrothermal synthesis [106], and chemical vapor condensation [107], typically follow the bottom–up approach. These methods allow for greater control over particle size and shape by building nanoparticles from the molecular or atomic level [100]. In recent years, green synthesis methods have gained attention as an alternative, offering a more environmentally friendly and cost-effective way to produce nanoparticles [108, 109]. These techniques utilize biological agents to mediate the synthesis of nanoparticles [110]. In this context, the synthesis of TiO_2_ NPs typically involves the reduction of titanium precursors using natural reducing agents, which also function as capping agents to reduce agglomeration [100]. A variety of studies have demonstrated the successful synthesis of TiO_2_ NPs using plant extracts. For example, the synthesis of TiO_2_ NPs has been reported using extracts from *Phyllanthus niruri* leaves [111], *Annona squamosa* peels [112], *Kniphofia foliosa* roots [113], and *Caesalpinia pulcherrima* flowers [114]. Additionally, microorganisms, including fungi such as *A. flavus* [115], *F. oxysporum* [116], and *Trichoderma citrinoviride* [117]; bacteria such as *Aeromonas hydrophila* [118], *Streptomyces* sp. HC1 [119], and *Bacillus cereus* [120]; and yeasts such as *Saccharomyces cerevisiae* [121], have been used for the biosynthesis of TiO_2_ NPs.

### Scaffolds and dressings containing TiO_2_ NPs for wound healing applications

While enhancing wound healing remains an ongoing challenge, several studies in recent years have developed various biomaterial-based scaffolds and dressings loaded with TiO_2_ NPs for applications in skin tissue engineering and wound healing. As summarized in Table 1, promising efforts have utilized different biomaterials and fabrication techniques to create structures containing TiO_2_ NPs. These studies demonstrate the potential of nanocomposite scaffolds and dressings in accelerating wound healing and promoting skin regeneration.

**Table 1:** Scaffolds and dressings containing TiO_2_ NPs for wound healing applications

Biomaterials	Scaffold/wound dressing type	Cell type (*in vitro* assay(s))	Animal model (wound type)	Main outcomes	Refs
Chitosan-CeO_2_/TiO_2_ NPs/PCL	Nanohybrid scaffolds prepared by casting method	NIH3T3 fibroblast cell line and human keratinocyte cell line HaCaT (MTT assay)	Male albino Wistar rats (excisional wounds)	- Suitable physicochemical properties—Higher tensile strength of Chitasan-CeO_2_/TiO_2_ NPs/PCL scaffolds compared to chitosan-CeO_2_ scaffolds—Good cell compatibility—Antibacterial activity against *E. coli* and *S. aureus*—Accelerated wound closure—Improved re-epithelialization, cell proliferation, and granulation tissue formation	[122]
Carboxymethyl chitosan/MSC-derived exosomes encapsulated with chitosan NPs loaded with BG and TiO_2_ NPs	Hydrogel dressings	HUVECs (CCK-8 and scratch assays), L-929 cells (CCK-8 assay), and RAW264.7 murine macrophage line (Inflammation assay)	Male C57BL/c mice (full-thickness skin defects), male db/db diabetic mice (full-thickness diabetic wounds), and male C57 mice (burn wounds)	- Suitable physicochemical properties—Increased viability of L-929 cells and HUVECs—Enhanced HUVECs migration in scratch assay—Increased expression of VEGFA and VEGFR2, demonstrating the capacity of the prepared hydrogel to induce angiogenesis in endothelial cells—Decreased secretion of pro-inflammatory factors (TNF-α, IL-1β, and IL-6) and increased secretion of anti-inflammatory cytokines (TGF-β and IL-10) in RAW 264.7 cells—Antibacterial activity against *E. coli* and *S. aureus*—Remarkably accelerated the wound healing process by enhancing vascularization of granulation tissues and promoting collagen deposition in acute full-thickness wounds, chronic diabetic wounds, and burn wounds—Increased expression of α-SMA and CD31 proteins *in vivo*, demonstrating newly formed blood vessels—Decreased TNF-α, IL-1β, and IL-6, and increased IL-10 during the diabetic wound healing process	[123]
PCL/TiO_2_ NPs	Electrospun nanofibrous scaffolds	Foreskin fibroblast cells (MTT assay and DAPI staining)	-	- Suitable physical properties—Enhanced tensile strength and elongation at break due to the incorporation of TiO_2_ NPs—Good biocompatibility and cell attachment—Antibacterial properties against *E. coli* and *S. aureus*	[124]
Chitosan or alginate/Ag NPs/TiO_2_ NPs	3D-printed hydrogels	Human fibroblasts (resazurin assay)	-	- Appropriate physicochemical and mechanical properties—Better biocompatibility of chitosan-based hydrogels compared to alginate-based ones—Cytotoxicity of Ag NPs in a dose and time-dependent manner—No significant cytotoxicity due to the presence of TiO_2_ NPs—Antibacterial activity against *Pseudomonas aeruginosa* and *S. aureus*	[125]
Dopamine-modified hyaluronic acid/gelatin/tyrosinase/PHMB/TiO_2_ NPs	Sponge dressing prepared by freeze drying and vacuum drying	HPMECs (live/dead staining and MTT assay) and mouse embryonic fibroblast 3 T3-L1 cell line (live/dead staining, MTT assay, and scratch assay)	Male SD rats (full-thickness burn wounds infected with *E. coli*–*S. aureus* suspension)	- High mechanical properties—Good liquid absorption capacity—Good wet adhesion—Antibacterial properties against *S. aureus*, *E. coli*, and *Pseudomonas aeruginosa*—ROS scavenging activity—High cytocompatibility—Promoted cell proliferation and migration *in vitro*—High hemocompatibility—Significantly reduced liver bleeding in rats—Accelerated the healing process of infected full-thickness burn wounds by inhibiting bacterial growth, accelerating skin tissue re-epithelialization, collagen deposition, and angiogenesis, as well as regulating the expression of CD31, IL-10, and TNF-α.	[126]
Chitosan/TiO_2_ NPs	Gel	-	Diabetic male albino Wistar rats (excisional wounds)	- Suitable pH, spreadability, and viscosity for wound healing applications—Desirable rheological properties—Enhanced wound contraction and accelerated wound closure—Enhanced granulation tissue formation—Promoted re-epithelialization—Exhibited anti-inflammatory effects *in vivo*—Improved angiogenesis—Promoted hair follicles regeneration	[127]
Carbopol®934/TiO_2_ NPs	Hydrogel	-	Male Swiss albino mice (excision wounds)	- Accelerated wound closure rate—Anti-inflammatory effects *in vivo*—Promoted angiogenesis—Enhanced re-epithelialization—Increased hydroxyproline content (an indicator of increased collagen deposition)	[128]
PVA/chitosan-g-poly (N-vinyl imidazole)/TiO_2_ NPs/curcumin	Electrospun nanofibers	Human fibroblasts (MTT assay)	Adult male rats (full-thickness excisional wounds)	- Suitable physicochemical properties—Excellent mechanical properties—Excellent hydrolytic degradation stability—Antibacterial activity against *E. coli* and *S. aureus*—No cytotoxic effects on normal fibroblast cells—*In vitro* antioxidant activity due to the presence of curcumin and TiO_2_ NPs—Enhanced wound closure rate—Promoted re-epithelialization—Decreased inflammatory cells infiltration—Enhanced skin appendages regeneration—Improved collagen distribution and maturation	[129]
Heparin-PVA/TiO_2_ NPs	Hydrogel	HFFF2 fibroblasts (MTT assay)	Male Kunming mice (full-thickness excision wounds)	- Suitable physicochemical properties—Improved tensile strength, elongation to break, and Young’s modulus due the presence of TiO_2_ NPs—Antibacterial activity against *E. coli* and *S. aureus*—Enhanced cell proliferation *in vitro*—Accelerated wound closure—Promoted the wound healing process	[130]
GelMA/xanthan gum/TiO_2_ NPs/N-halamine	3D-printed dressing	HEK 293 T cells (CCK-8 assay)	BALB/c mice (excisional wounds)	- Antibacterial activity against *S. aureus* and *E. coli* O157:H7—Biofilm-controlling effects against *S. aureus* and *E. coli* O157:H7—Good biocompatibility—Accelerated wound healing	[131]
Chitosan/carboxymethyl guar gum/TiO_2_ NPs	Freeze-dried dressings	C3H10T1/2 mouse cells (MTT and scratch assays)	-	- Appropriate physicochemical properties—Enhanced compressive strength due to the presence of TiO_2_ NPs—Antimicrobial activity against *E. Coli*, *S. Aureus*, and *A. Niger*—Increased viability of C3H10T1/2 cells by increasing the concentration of TiO_2_ NPs up to 2 wt. %—Promoted migration of C3H10T1/2 cells in scratch assay	[132]
Chitosan/PPG/TiO_2_ NPs	Hydrogel composite film	-	-	- Suitable physicochemical properties—Enhanced tensile strength and elongation at break due to the presence of TiO_2_ NPs—Antimicrobial activity against *S. aureus*, *E. coli* and *Candida lipolytica*—Formation of apatite on the surface of the hydrogel, demonstrating its biomineralization ability	[133]
Gellan gum/TiO_2_ NPs	Films prepared by casting	3 T3 mouse fibroblast cells (acridine orange/propidium iodide staining, MTT assay, and scratch assay)	SD rats (full-thickness excision wounds)	- Suitable physicochemical properties—Antibacterial activity against *S. aureus* and *E. coli*—Enhanced Young’s modulus and tensile strength due to the presence of TiO_2_ NPs—Slightly decreased toughness and elongation-at-break due to the presence of TiO_2_ NPs—Promoted cell proliferation *in vitro*—Enhanced cell migration in scratch assay—Enhanced wound closure rate	[25, 134]
Gelatin/TiO_2_ NPs	Gel	-	Male rats (full-thickness excision wounds infected with MRSA)	- Antibacterial activity against *S. aureus*—Reduced total bacterial count *in vivo*—Promoted wound contraction—Accelerated re-epithelialization—Increased neovascularization—Stimulated proliferation of fibroblasts—*In vivo* anti-inflammatory effects—Increased hydroxyproline content (an indicator of increased collagen deposition)	[135]
TiO_2_ NPs/gelatin	Gel	L929 murine fibroblast cells (MTT assay)	Male BALB/c mice (full-thickness burn wounds)	- Antibacterial activity against *S. aureus* and *Pseudomonas aeruginosa*—No significant *in vitro* cytotoxicity against L929 cells when using TiO_2_ NPs/gelatin containing up to 1 mg/mL of TiO₂ NP—Promoted wound contraction—*In vivo* anti-inflammatory effects—Increased angiogenesis—Increased fibroblasts proliferation *in vivo*—Enhanced granulation tissue formation—Promoted re-epithelialization	[136]
Sodium alginate/PVA/TiO_2_ NPs/curcumin	Scaffolds prepared by gel casting method	-	-	- Antibacterial activity against *Bacillus subtilis* and *Klebsiella pneumoniae*	[137]
Polyurethane/TiO_2_ NPs/chitosan NPs	Membranes prepared by the dry/wet phase inversion method	Human fibroblast cells (MTT assay)	-	- Suitable physicochemical properties—Antibacterial properties against *Pseudomonas aeruginosa* and *S. aureus*—High biocompatibility—Proper ultimate tensile strength and elongation at break values	[138]
Chitin/silk fibroin/TiO_2_ NPs	Freeze-dried scaffolds	HFFF2 fibroblasts (MTT assay and DAPI staining)	-	- High porosity—Excellent water uptake ability—Appropriate biodegradation rate—Good blood clotting ability—Antimicrobial activity against *S. auresus*, *E. coli* and *C. albicans*—Good cytocompatibility	[139]
Bacterial cellulose/TiO_2_ NPs	Nanocomposite films	-	BALB/c mice (partial-thickness burn wounds)	- Appropriate physicochemical properties—Antibacterial activity against *S. aureus* and *E. coli*—Accelerated the wound healing process—Promoted re-epithelialization—Improved angiogenesis	[140]
PVA/Pluronic F127/PEI/TiO_2_ NPs	Electrospun nanofibers	Fibroblast cells (neutral red assay)	-	- Tensile strength increased due to the incorporation of TiO_2_ NPs—The presence of TiO_2_ NPs reduced cytotoxicity of PVA/Pluronic F127/PEI blend—Antibacterial activity against *Pseudomonas aeruginosa*, *Salmonella typhi*, and *E. coli*	[141]
Gentamicin-loaded PCL/TiO_2_ NPs	Electrospun membranes	-	-	- Increased the degree of crystallinity due to the incorporation of TiO_2_ NPs—Decreased diameter of fibers due to the incorporation of TiO_2_ NPs—Antibacterial activity against MRSA	[142]
Bacterial cellulose/TiO_2_ NPs	Films prepared by the casting method	Animal fibroblast cells (MTT assay)	-	- Low to moderate level of Ti^4+^ release—Antibacterial activity against *E. coli* through ROS generation and membrane stress induced by TiO_2_ NPs—Excellent cell growth and proliferation	[143]
Collagen-coated PCL/TiO_2_ NPs	Electrospun fibers	-	-	- Nanofiber diameter ranged from 200 to 800 nm—Collagen coating increased hydrophilicity—The presence of TiO_2_ NPs increased the tensile strength, while the collagen coating decreased it.	[144]

*TiO*_*2*_  *NPs* Titanium dioxide nanoparticles, *CeO*_*2*_ Cerium oxide, *PCL* Polycaprolactone, *MTT* 3-(4,5-Dimethylthiazol-2-yl)-2,5-Diphenyltetrazolium Bromide, *MSC* mesenchymal stem cell, *BG* Bioactive glass, *HUVECs* Human umbilical vein endothelial cells, *CCK-8* cell counting kit-8, *VEGFA* Vascular endothelial growth factor A, *VEGFR2* VEGF receptor 2, *TNF-α* Tumour necrosis factor alpha, *IL-1β* Interleukin 1 beta, *IL-6* Interleukin 6, *TGF-β* Transforming growth factor beta; *IL-10* Interleukin 10, *α-SMA* alpha-smooth muscle actin; *CD31* cluster of differentiation 31, *DAPI* 4′,6-diamidino-2-phenylindole, *Ag NPs* Silver nanoparticles, *PHMB* Polyhexamethylene biguanide, *ROS* Reactive oxygen species, *HPMECs* human pulmonary microvascular endothelial cells, *SD* rats Sprague–Dawley rats, *PVA* Polyvinyl alcohol, *GelMA* Gelatin methacryloyl, *HEK 293 T* Human embryonic kidney 293 T, *PPG* Polypropylene glycol, *MRSA* Methicillin-resistant *Staphylococcus aureus*, *PEI* polyethylenimine

#### Electrospun fibers

Electrospun fibers are ultrafine fibers produced through electrospinning. In this technique, a high voltage is applied to a polymer solution or melt, causing the solution to form a jet that is then stretched and solidified into fibers as it travels towards a grounded collector. This process allows for the production of nanofibers with a high surface area-to-volume ratio, high porosity, and controlled fiber alignment [145–147]. By controlling parameters such as polymer concentration, solvent choice, applied voltage, and flow rate, the properties of electrospun scaffolds can be tailored to meet specific requirements [145]. Electrospun scaffolds can mimic the ECM [148]. They can facilitate cell adhesion, proliferation, and migration [5]. These scaffolds also provide physical support, protecting the wound and supporting the regeneration process. The porous structure of electrospun scaffolds facilitates the exchange of oxygen and nutrients, which is crucial in the wound healing process [149, 150]. Additionally, the large surface area of electrospun nanofibers allows for the incorporation of bioactive agents and nanomaterials that can further enhance wound healing. Electrospun nanofibers incorporating bioactive agents, such as growth factors or antimicrobials, can promote wound healing by providing localized and sustained release of therapeutic molecules [151, 152]. Nanomaterials can also be incorporated into electrospun nanofibers to provide various functionalities, such as antimicrobial properties [153], enhanced mechanical strength [154], or improved angiogenesis [155].

**Figure 4:**
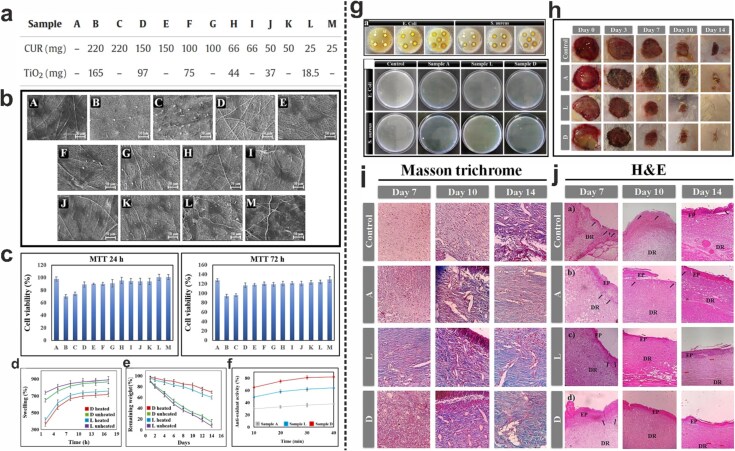
(**a**) Curcumin and TiO_2_ NP contents of PVA/CS-g-PNVIM-based wound dressings (note: All dressings were prepared using 0.3 g of CS-PNVIM and 10 g of PVA (8% w/w)). (**b**) SEM images of human fibroblast adhesion after 24 hours of culture on each sample. (**c**) Viability of fibroblasts cultured on each sample for 24 and 72 hours. (**d**) Swelling ratio of heated and unheated D and L dressings. (**e**) Degradation of heated and unheated D and L dressings. (**f**) Antioxidant activity of A, D, and L dressings. (**g**) Antibacterial activity of the prepared dressings against *E. coli* and *S. aureus* using the disc diffusion assay. The colonies of *E. coli* and *S. aureus* after treatment with samples A, L, and D are also shown. (**h**) Macroscopic images of the healing wounds on days 0, 3, 7, 10, and 14 treated with A, D, and L dressings. The wounds of the control group were treated with sterile cotton gauze. (**i** & **j**) Masson's trichrome and H&E staining of the control group and wounds treated with A, L, and D dressings for 7, 10, and 14 days. Inflammatory cells are indicated by black arrows, while red arrows indicate hair follicles. EP and DR refer to epidermis and dermis, respectively. Adapted with permission from Elsevier [129]

Some studies have focused on designing and developing electrospun constructs incorporated with TiO_2_ NPs for wound healing applications. For instance, Motasadizadeh et al. focused on the development and evaluation of PVA/chitosan-g-poly(N-vinyl imidazole) (PVA/CS-g-PNVIM) wound dressings containing TiO_2_ NPs/curcumin (CUR) for enhanced wound healing (Figure 4). They emphasized the multifunctional properties of these dressings and their potential for practical application in wound management. The mechanical, structural, and biological properties of the nanofiber dressings were thoroughly investigated using various techniques. It was found that the dressings displayed excellent mechanical and hydrolytic degradation stability, indicating their potential durability in a wound environment. DPPH assay indicated that the antioxidant activity of CUR-containing nanofibers increased over time, with nanofibers containing higher concentrations of CUR showing greater antioxidant potency, reaching up to 82% after 40 minutes. Additionally, the CS-g-PNVIM-based nanofibers demonstrated remarkable antibacterial activity against both *E. coli* and *S. aureus*. Importantly, these nanofibers did not exhibit cytotoxicity towards normal fibroblast cells, ensuring their biocompatibility. *In vivo* studies further supported the efficacy of CS-g-PNVIM-based nanofibers in promoting wound closure and epithelial layer formation, demonstrating their effectiveness in supporting the wound healing process and tissue regeneration. The dressings, particularly those containing TiO_2_ NPs and CUR, showed improved wound healing effects with reduced inflammatory responses, increased collagen distribution and maturation, and enhanced re-epithelialization [129].

#### 3D-printed scaffolds/dressings

3D printing, also known as additive manufacturing, is a process that creates three-dimensional objects by layering material in a controlled manner based on a digital design [156]. This technology has diverse applications in fields such as aerospace [157], automotive industry [158], healthcare [159], architecture [160], and manufacturing [161], enabling rapid prototyping, customization, and the creation of complex geometries. In biomedical applications, 3D printing facilitates the fabrication of complex structures with precise control over geometry and internal architecture, allowing the creation of patient-specific constructs for tissue regeneration [162, 163]. In wound healing applications, 3D-printed constructs offer several advantages. They can be designed to match the specific shape and size of the wound, effectively covering the wound area [164]. The porous structure of these constructs allows for the infiltration of cells and nutrients, promoting tissue regeneration [165]. Furthermore, 3D printing enables the incorporation of drugs, growth factors, and bioactive agents into the constructs, which can significantly enhance wound healing [166–168]. The ability to precisely control the composition and spatial distribution of these components within the 3D-printed constructs allows for the development of tailored and targeted therapeutic approaches [168, 169]. 3D-printed scaffolds/dressings incorporating nanoparticles with specific therapeutic properties have been shown to effectively enhance wound healing [168, 170, 171].

**Figure 5:**
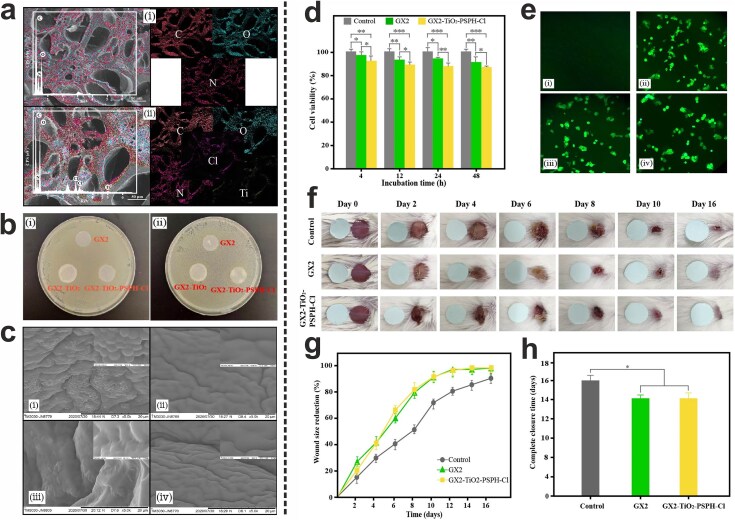
(**a**) SEM images and element mapping of GX2 (i) and GX2-TiO_2_-PSPH-Cl (ii) wound dressings. (**b**) Antibacterial activity of GX2, GX2-TiO_2_, and GX2-TiO_2_-PSPH-Cl dressings against *S. aureus* (i) and *E. coli* O157:H7 (ii), using the disc diffusion assay. (**c**) SEM images showcasing the biofilm-controlling effects of GX2 dressing against *S. aureus* (i) and *E. coli* O157:H7 (iii), and GX2-TiO_2_-PSPH-Cl dressing against *S. aureus* (ii) and *E. coli* O157:H7 (iv). (**d**) CCK-8 assay results demonstrating the viability of human embryonic kidney 293 T (HEK 293 T) cells after incubation for 4, 12, 24 and 48 hours (^*^P ≤ 0.05, ^*^^*^P ≤ 0.01, and ^*^^*^^*^P ≤ 0.001). (**e**) Morphology of HEK 293 T cells after incubation for 24 hours. i-iv represent negative control, control, GX2, and GX2-TiO_2_-PSPH-Cl groups, respectively. (**f**) The effects of gauze, GX2, and GX2-TiO_2_-PSPH-Cl dressings on the excisional wounds in BALB/c mice. (**g** & **h**) Wound size reduction percentage and complete closure time of the excisional wounds treated with gauze, GX2, and GX2-TiO_2_-PSPH-Cl dressings (^*^P ≤ 0.05). (GX2: GelMA/xanthan gum dressing containing 2 wt% xanthan gum, GX2-TiO_2_: GelMA/xanthan gum/TiO_2_ NPs dressing, and GX2-TiO_2_-PSPH-Cl: GelMA/xanthan gum/TiO_2_ NPs/N-halamine dressing). Adapted with permission from Elsevier [131]

Yang et al. developed a 3D-printed wound dressing using a combination of gelatin methacrylate (GelMA) and xanthan gum as the base material, enhanced with N-halamine and TiO_2_ NPs for improved antibacterial properties. The prepared dressing exhibited an excellent swelling ratio and water uptake capacity. The introduction of TiO_2_ NPs via in-situ synthesis not only improved the UV stability of the N-halamines but also enhanced the antibacterial activity of the dressing against *S. aureus* and *E. coli* O157:H7. The bacterial biofilm test further indicated the ability of the 3D-printed dressing to inhibit biofilm formation. Additionally, the dressing exhibited good biocompatibility and significantly accelerated the healing of excisional wounds in BALB/c mice, suggesting that the developed 3D-printed dressing is a promising candidate for effective wound treatment (Figure 5) [131].

#### Casted films

Casted films are of interest in wound healing applications due to their versatility and efficacy. These films can serve as protective barriers, shielding wounds from external contaminants while providing an optimal environment for tissue regeneration. Moreover, they can be loaded with therapeutic agents, such as antibiotics, growth factors, and other bioactive agents to accelerate the healing process. Casted films offer significant advantages, such as simple fabrication, versatility in using a variety of biomaterials, and the accessibility of relatively inexpensive equipment, making them efficient and cost-effective candidates for wound healing applications. The fabrication process typically involves a casting process in which the desired materials are mixed and poured into molds, allowing for the customization of specific shapes and sizes [172–174].

**Figure 6:**
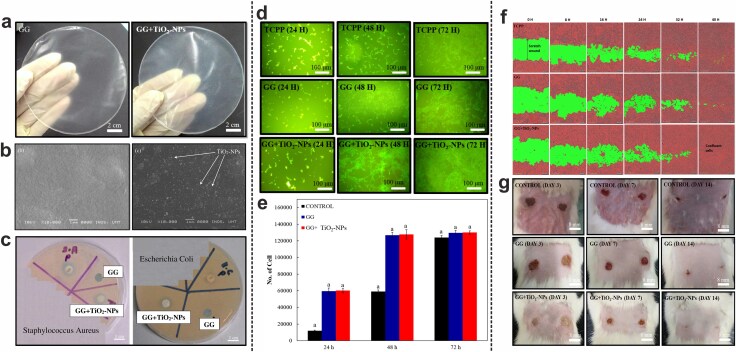
(**a**) Macroscopic images of gellan gum (GG) and gellan gum/TiO_2_ NPs (GG + TiO_2_-NPs) dressings. (**b**) SEM micrographs of GG and GG + TiO_2_-NPs films. (**c**) Antibacterial activity of GG and GG + TiO_2_-NPs films against *S. aureus* and *E. coli*. Penicillin (P) antibiotic was used as the positive control. (**d**) Fluorescence microscope images captured after 24, 48, and 72 hours of cell culture on GG and GG + TiO_2_-NPs samples. TCPP substrates were used as the control group. (**e**) Number of fibroblasts in different groups after 24, 48, and 72 hours of culture. (**f**) Scratch assay depicting the migration of fibroblasts on TCPP substrate, GG film, and GG + TiO_2_-NPs film at 8-hour intervals. Scale bars indicate 300 μm. (**g**) The effects of GG and GG + TiO_2_-NPs dressings on full-thickness wounds in Sprague Dawley rats after 3, 7, and 14 days of treatment. The control group received no treatment. Adapted with permission from Elsevier [25]

Ismail et al. conducted a study aiming to investigate the potential of TiO_2_ NPs-incorporated gellan gum (GG + TiO_2_-NPs) film as an antibacterial wound dressing prepared by an evaporative casting technique. The GG + TiO_2_-NPs film exhibited favorable physicochemical and mechanical properties suitable for wound healing applications. Additionally, the prepared GG + TiO_2_-NPs film demonstrated significant antibacterial properties against both *S. aureus* and *E. coli*, with no such effects observed for pure gellan gum (GG) film. *In vitro* tests on 3 T3 mouse fibroblast cells showed that cell spreading increased over time for both films, with the GG + TiO_2_-NPs film demonstrating the highest number of adhered cells compared to the GG film and tissue culture polystyrene plate (TCPP). After 72 hours, the cell count for the GG + TiO2-NPs film reached approximately 127 733 cells/well, indicating superior cell growth compared to the GG film and TCPP, with ~126 733 cells/well and 123 844 cells/well, respectively. The enhanced cell compatibility and proliferation were attributed to the surface roughness of the GG + TiO_2_-NPs film, affecting protein interactions and facilitating cell adhesion and proliferation. Furthermore, the scratch assay showed that the GG + TiO_2_-NPs film promoted more effective closure of scratch wounds compared to pure GG film and TCPP, demonstrating complete closure after 40 hours. *In vivo* studies on Sprague Dawley rats compared the efficacy of pure GG film, GG + TiO_2_-NPs film, and a negative control group (which received no treatments) in the treatment of full-thickness excision wounds. The GG + TiO_2_-NPs film showed the highest wound closure rate by day 14 of treatment. Quantitative analysis revealed that on Day 14 of treatment, almost complete healing (92%) was seen in the GG + TiO_2_-NPs group, outperforming the GG (80%) and control (75%) groups, showcasing the advantageous effects of TiO_2_ NPs in accelerating wound healing by inhibiting microbial infections and supporting tissue regeneration (Figure 6) [25].

#### Other types of scaffolds/dressings

Other types of scaffolds and dressings incorporating TiO_2_ NPs have also been reported to efficiently promote the healing of various wound types *in vivo*. For instance, Li et al. developed a multifunctional hybrid sponge dressing composed of dopamine-modified hyaluronic acid, gelatin, polyhexamethylene biguanide (PHMB), and TiO_2_ NPs for the effective treatment of infected burn wounds. The prepared dressing exhibited robust mechanical properties, effective hemostasis, broad-spectrum antibacterial activity, ROS scavenging, and biocompatibility. *In vivo* studies on Sprague–Dawley rats with full-thickness burn wounds infected with an *E. coli*–*S. aureus* suspension demonstrated significantly accelerated wound healing. The dressing effectively inhibited bacterial growth, enhanced re-epithelialization, promoted collagen deposition, increased angiogenesis, and regulated the expression of CD31, IL-10, and TNF-α, showcasing its potential as an advanced wound care solution [126]. Ahmad et al. utilized *Ocimum sanctum* leaf extract as a reducing agent to synthesize TiO_2_ NPs, which were then incorporated into a 2% chitosan gel for topical application on diabetic wounds. The chitosan gel containing TiO_2_ NPs displayed thixotropic properties and pseudoplastic behavior, which are desirable for topical applications. *In vivo* studies on diabetic Wistar rats with excision wounds demonstrated that the TiO_2_ NPs-containing chitosan gel had excellent wound-healing efficacy, as evidenced by faster wound contraction and re-epithelialization. Histopathological examinations further supported the efficacy of the developed formulation. The results revealed that the TiO_2_ NPs-containing chitosan gel significantly reduced inflammation, enhanced keratinization, and promoted the formation of hair follicles, granulation tissue, and blood vessels [127]. In a study by Khalid et al., the effectiveness of a bacterial cellulose/TiO_2_ NPs nanocomposite was evaluated for the treatment of partial-thickness burn wounds in BALB/c mice. The prepared nanocomposite exhibited antibacterial activity against *E. coli* and *S. aureus*. *In vivo* studies demonstrated that wounds treated with the bacterial cellulose/TiO_2_ NPs nanocomposite showed accelerated closure compared to wounds treated with pure bacterial cellulose and untreated wounds, indicating the effectiveness of the incorporated TiO_2_ NPs. Histological analysis revealed enhanced re-epithelialization, the formation of healthy granulation tissue, and improved angiogenesis in wounds treated with the bacterial cellulose/TiO_2_ NPs nanocomposite [140].

### Bi-layered scaffolds/dressings containing TiO_2_ NPs for wound healing

**Figure 7:**
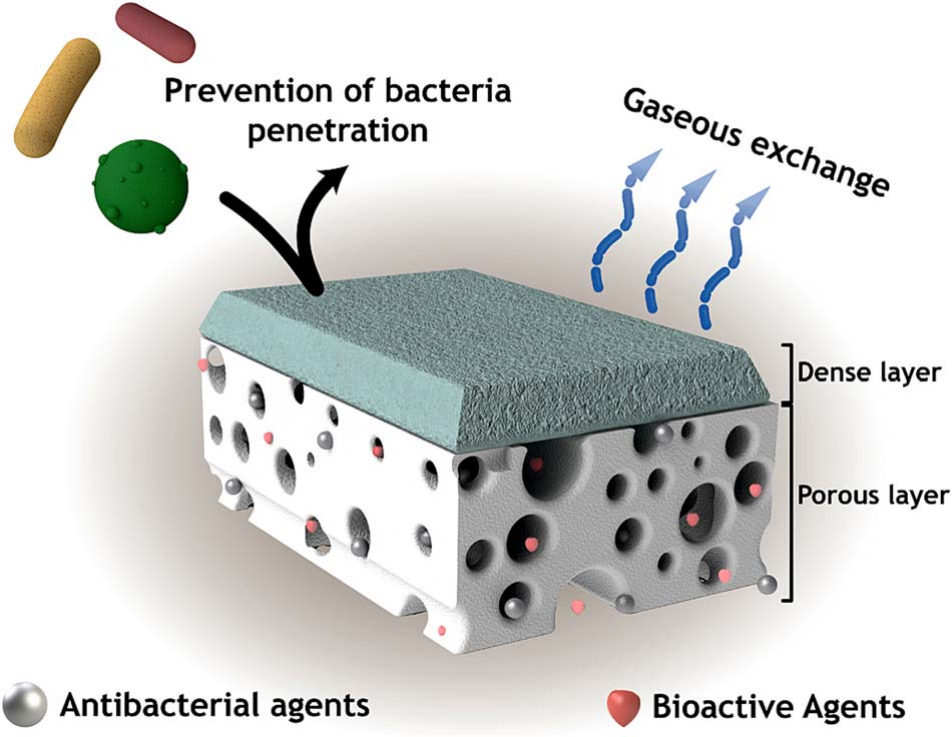
A bi-layered scaffold/dressing with properties suitable for wound healing applications. Adapted and reprinted with permission from Elsevier [177]

Injuries and chronic wounds can affect not only the main layers of the skin but also deeper tissues, making their treatment complex [49]. The epidermis, primarily composed of layers of keratinocytes, provides a protective barrier for the underlying tissues [175]. The dermis layer is characterized by a low cell density and a high amount of ECM. It provides structural support and elasticity to the skin. The regeneration of the epidermis and dermis involves different mechanisms and complexities [176]. Therefore, it is necessary to develop new approaches that take into account the unique properties of each layer. One promising method for wound treatment is the use of bi-layered scaffolds/dressings. Bi-layered constructs, consisting of a dense top layer and a porous underlying layer, have been found to be more suitable for the treatment of full-thickness skin wounds. The top layer acts as a barrier, preventing bacterial infections and water loss, while still allowing for gaseous exchange [177, 178]. The underlying porous layer promotes the proliferation and migration of fibroblasts, provides space for ECM synthesis, supports angiogenesis, absorbs fluids, and facilitates tissue nutrition [179, 180]. The biological properties of these scaffolds/dressings can be further enhanced by incorporating bioactive nanomaterials. Previous studies have demonstrated significant improvements in properties such as angiogenic activity, anti-inflammatory effects, and antibacterial activity when bioactive nanomaterials are added. Such scaffolds/dressings can more effectively support the healing process and contribute to superior skin regeneration outcomes [181–183]. Figure 7 depicts a bi-layered scaffold/dressing suitable for wound healing applications.

**Figure 8:**
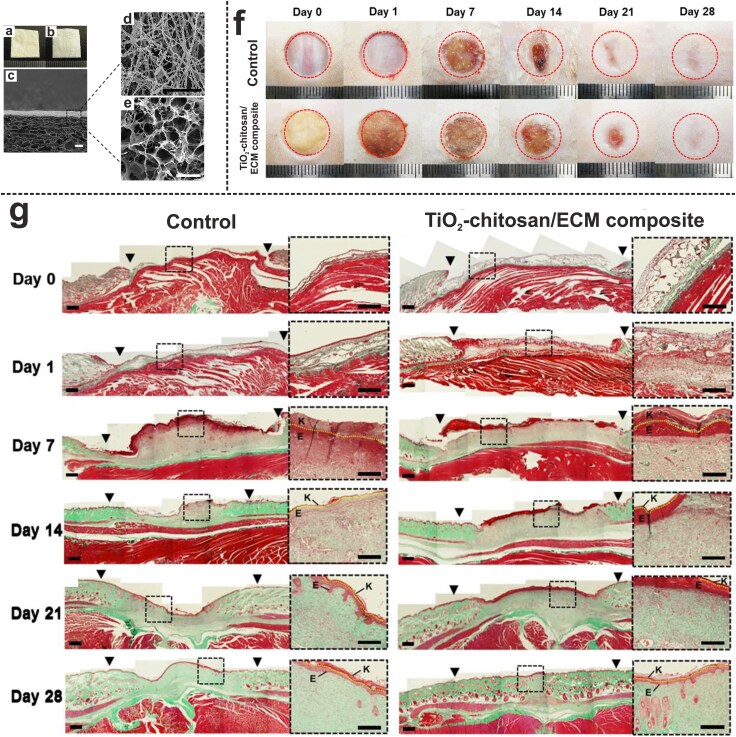
(**a**) Macroscopic top view of the bilayer dressing (TiO_2_ NPs-incorporated chitosan layer). (**b**) Macroscopic bottom view of the bilayer dressing (ECM layer). (**c**) SEM micrograph of the bilayer dressing (scale bar represents 200 μm). (**d**) SEM micrograph of the electrospun top layer (scale bar represents 5 μm). (**e**) SEM micrograph of the underlying layer (scale bar represents 200 μm). (**f**) Promotion of the healing process of full-thickness wounds in Sprague–Dawley rats using the bilayer composite dressing. (**g**) Histological micrographs of the healing wounds (Gomori’s trichrome staining). Keratin and epidermal layers are shown by the letters K and E, respectively, with the yellow-dotted lines separating layers. The initial wound areas are indicated by ▼ (scale bars represent 200 μm). Adapted with permission from [185]

Rezk et al. developed a bi-layered electrospun membrane for wound healing applications. The top layer, consisting of PCL, provided mechanical strength and formed a hydrophobic barrier against the external environment. The underlying layer, made of polydioxanone loaded with TiO_2_ NPs and tetracycline, served as a delivery system with antibacterial properties. Electrospinning ensured the uniform distribution of TiO_2_ NPs and tetracycline within the nanofibers. Characterization of the membrane revealed appropriate morphology, fiber diameter, mechanical properties, and surface wettability. *In vitro* drug release studies showed an initial burst release of tetracycline followed by sustained release over 4 days. The nanofibers exhibited excellent antibacterial properties against both *S. aureus* and *E. coli*. Furthermore, the bi-layered membrane was non-toxic to NIH3T3-E1 fibroblasts. Although the results demonstrated the potential of the prepared membrane for wound healing applications, the study did not include any *in vivo* experiments to further validate its effectiveness [184]. Woo et al. designed a bilayer composite wound dressing comprising an upper layer made of electrospun chitosan membrane incorporated with TiO_2_ NPs, and an underlying layer of human adipose-derived ECM sheet to enhance the healing of full-thickness wounds in Sprague–Dawley rats (Figure 8). The dense, fibrous top layer provides antibacterial protection, while the sponge-like underlying layer promotes tissue regeneration. The bacterial penetration test showed a 33.9% reduction in viable *E. coli* and a 69.58% reduction in viable *S. aureus*, indicating that the bilayer composite more effectively inhibited the penetration of *S. aureus*. *In vivo* tests demonstrated that the bilayer composite adhered well to wounds and absorbed exudate effectively. By Day 14 of the healing process, wounds treated with the bilayer composite exhibited significantly reduced sizes (52.0%) compared to control wounds (39.9%). By Day 28, nearly complete healing was observed in both groups, with remaining wound areas of 17.4% for treated and 23.1% for control wounds. Histological studies revealed that by Day 7, a keratin layer had formed in the treated wounds, whereas the control wounds lacked a clear epidermal layer. The bilayer composite degraded rapidly, with ECM sheets mostly replaced by newly secreted collagen by week 3. By Day 28, the regenerated skin featured numerous dermal appendages. Additionally, the treated wounds showed a higher number of CD31-expressing endothelial cells and a higher density of microvessels at weeks 2 and 3, indicating enhanced blood vessel formation. The study concluded that the prepared bilayer composite could mimic the structural and functional properties of normal skin, offering a promising solution for effective wound care [185].

### Modified TiO_2_ NPs for wound healing

Modified nanoparticles have shown remarkable potential in diverse biomedical applications, with doped nanoparticles being a prominent area of study. Doping nanoparticles involves incorporating impurities or foreign atoms into the crystal lattice of nanoparticles to modify their various properties, such as physical, chemical, optical, electrical, and biological characteristics [186, 187]. By introducing specific dopants into the TiO_2_ NPs, their properties can be finely tuned to enhance their performance in various biomedical applications [22]. Scaffolds and dressings containing doped TiO_2_ NPs have demonstrated promising outcomes in the treatment of skin wounds. In a study by Wang et al., Ag-doped TiO_2_ NPs were synthesized using a chemical reduction method. Initially, TiO_2_ NPs were dispersed in deionized water, and sodium dodecyl benzene sulfonate was added to form a suspension. Silver nitrate (AgNO_3_) was dissolved in deionized water, mixed with aqueous ammonia to prepare a silver-ammonia solution, which was then added to the TiO_2_ suspension along with glucose as a reducing agent. This mixture was stirred vigorously for 24 hours at 35°C. Subsequently, the resulting Ag/TiO_2_ suspension was centrifuged, washed, and dried to obtain Ag-doped TiO_2_ samples with varying silver content of 0.2%, 0.5%, 1%, 2%, and 5%. The researchers then developed antibacterial hydrogels by embedding the prepared Ag-doped TiO_2_ NPs in PVA matrices. Under 660 nm visible light irradiation, these hydrogels exhibited remarkable antibacterial activity against *S. aureus* and *E. coli* bacteria due to ROS release. Additionally, PVA hydrogels incorporated with Ag-doped TiO_2_ NPs, containing 0.5% silver, showed good biocompatibility and effectively prevented wound infections *in vivo*, significantly accelerating the healing of *S. aureus*-infected wounds in male Kunming rats. Moreover, no appreciable toxicity to vital organs, including the heart, liver, spleen, lung, and kidney, was reported [188].

Nanoparticles can undergo surface modifications to enhance their physicochemical and biological properties [189, 190]. Niranjan et al. synthesized curcumin-tagged TiO_2_ NPs through a two-step process. First, they prepared TiO_2_ NPs by adding titanium tetraisopropoxide to a solution of 1 M aqueous nitric acid, followed by agitation for 2 hours and pH adjustment to 3 using a diluted solution of 1 M NaOH. The resulting TiO_2_ NPs were centrifuged, rinsed with distilled water, dried at 100°C for 1 hour, and then calcined at 450°C for 3 hours. In the second step, TiO_2_ NPs were dispersed in distilled water and stirred, while curcumin was dissolved in dichloromethane and stirred separately. The curcumin solution was then added dropwise to the TiO_2_ NPs/water mixture and stirred for 24 hours, resulting in a color change from yellow to orange. The resulting mixture was centrifuged, the supernatant was discarded, and the final product was dried at 60°C, yielding curcumin-tagged TiO_2_ NPs. The attachment of curcumin on the surface of TiO_2_ NPs was confirmed by Fourier-transform infrared spectroscopy (FTIR) analysis. The researchers then developed a PVA/sodium alginate patch incorporated with the synthesized curcumin-tagged TiO_2_ NPs for wound healing applications. The *in vitro* drug release test over 25 days showed an initial burst release of curcumin in the first 5 days, followed by a gradual release. The prepared patch showed antibacterial properties against *Bacillus subtilis*, *S. aureus*, *E. coli*, and *P. aeruginosa*, suggesting its effectiveness against both Gram-positive and Gram-negative bacteria. The patch also enhanced the viability of NIH3T3 cells, suggesting its biocompatibility and potential for tissue regeneration. Furthermore, *in vivo* studies on Wistar albino rats with full-thickness wounds showed that the PVA/sodium alginate/TiO_2_ NPs-curcumin patch accelerated wound closure, increased hydroxyproline content, improved collagen deposition, and promoted re-epithelialization [191].

### Toxicity of TiO_2_ NPs

Nanoparticles have become invaluable across a range of fields, including medicine, the food industry, and cosmetics, due to their exceptional physicochemical properties [192–194]. The large surface area-to-volume ratios of nanoparticles provide them with enhanced reactivity compared to bulk materials, raising concerns about their potential hazards [195]. Consequently, the assessment of nanoparticle toxicity has become a critical area of study, given the limited knowledge available regarding their potential risks. The widespread use of nanoparticles highlights the importance of understanding their toxicity mechanisms to accurately predict their impact on human health [196, 197]. Particularly in biomedical applications, where nanoparticles play a significant role, a thorough assessment of their toxicity is paramount to ensure their safe and effective use [5]. In general, the toxicity of nanoparticles primarily depends on several factors, including the type of nanoparticle, concentration, duration of exposure, and specific physicochemical properties, such as size, shape, and surface characteristics [198, 199]. By delving deeper into the toxicity of nanoparticles, researchers can better address the concerns surrounding their potential adverse effects and pave the way for their responsible and beneficial utilization in biomedical applications.

There have been numerous reports highlighting the toxicity of metal oxide nanoparticles, including but not limited to zinc oxide (ZnO) [200, 201], copper oxide (CuO) [202], cerium oxide (CeO_2_) [203], iron oxide [204], magnesium oxide (MgO) [205], nickel oxide (NiO) [206], and cobalt oxide [207] nanoparticles. These studies have shed light on the potential risks associated with the use and exposure to these nanomaterials. TiO_2_ NPs can reach various parts of the body through exposure routes such as injection, gastrointestinal tract absorption, dermal deposition, and inhalation, highlighting the importance of assessing their toxicity [208]. Jin et al. conducted a study evaluating the cytotoxicity of weakly aggregated anatase TiO_2_ NPs in mouse fibroblast L929 cells. They investigated various concentrations of TiO_2_ NPs ranging from 3 to 600 μg/ml and observed significant morphological changes in L929 cells, including rounding and shrinking, with increasing nanoparticle concentration. Acridine orange staining revealed condensed fragmented chromatin and necrosis in nanoparticle-treated cells. Transmission electron microscopy (TEM) analysis showed an increased number of lysosomes, as well as damage to some organelles, in cells cultured in a medium containing 300 μg/ml TiO_2_ NPs. Additionally, concentrations exceeding 60 μg/ml led to increased oxidative stress. With the increase in the concentration of TiO_2_ NPs within the culture medium, there was a corresponding increase in the levels of ROS and lactate dehydrogenase, whereas cell viability and the cellular levels of glutathione and superoxide dismutase decreased. The findings suggest a dose-dependent induction of cytotoxic effects, including inhibition of cell proliferation, DNA damage, and disruption of redox homeostasis [209].

According to the literature, the cytotoxic effects of TiO_2_ NPs on skin cells have also been reported. To date, several studies have demonstrated the cytotoxicity of TiO_2_ NPs on keratinocytes and have reported various mechanisms for the corresponding toxic effects. For instance, Simon et al. conducted a study to investigate the mechanisms underlying the toxicity induced by TiO_2_ NPs on primary human foreskin keratinocytes. The findings revealed a dose-dependent decrease in cell proliferation, attributed to alterations in calcium homeostasis rather than cell death. Notably, the study elucidated that the surface chemistry of TiO_2_ NPs influences their toxicity, with naked particles inducing a more pronounced increase in intracellular calcium concentration compared to fluorescent dye-modified particles [210]. A study by Zhao et al. investigated the interaction between TiO_2_ NPs and human primary epidermal keratinocytes, with a focus on understanding the potential toxicological effects and the induction of autophagy. The cytotoxicity of TiO_2_ NPs was evaluated using a WST-8 assay, revealing a 15% reduction in cell viability after 24 hours of exposure to 10 μg/ml of TiO_2_ NPs. To investigate autophagy activation, the researchers used monodansylcadaverine staining and LC3 immunofluorescence. Both methods indicated a significant increase in autophagic vacuoles in cells treated with TiO_2_ NPs at a concentration of 10 μg/ml. TEM further confirmed the presence of autophagic vacuoles and other ultrastructural changes in keratinocytes, supporting the activation of autophagy. The researchers also found that even very low concentrations of TiO_2_ NPs (as low as 100 fg/ml) could induce autophagy in keratinocytes, while significant reductions in cell viability were observed at higher concentrations (50 pg/ml). The induction of autophagy varied with increasing TiO_2_ NPs concentrations, peaking at 50 ng/ml and decreasing thereafter. Conversely, cell viability decreased significantly with increasing TiO_2_ NPs concentrations. The findings suggest that autophagy possibly plays a protective role at lower concentrations but becomes overwhelmed at higher concentrations [211]. Gao et al. conducted a study on the cytotoxicity of TiO_2_ NPs on HaCaT cells. Their findings revealed significant adverse effects induced by TiO_2_ NPs, including reduced cell viability and damage to intracellular organelles. Additionally, TiO_2_ NPs triggered both early and late apoptosis in the cells, indicating a profound impact on cell survival mechanisms. Moreover, exposure to TiO_2_ NPs resulted in cell cycle arrest, further emphasizing their detrimental effects on cellular processes. The researchers also observed that TiO_2_ NPs were readily taken up by the cells, particularly noticeable at concentrations of 10 and 25 μg/ml, leading to an increase in cellular granularity. Furthermore, TiO_2_ NPs exhibited a high capacity to induce ROS generation, suggesting their potential to induce oxidative stress and cellular damage [212].

Keratinocytes are not the only skin cells that have been used to study the cytotoxic effects of TiO_2_ NPs. Pan et al. investigated the adverse effects of TiO_2_ NPs on human dermal fibroblasts, focusing specifically on rutile and anatase forms of TiO_2_ NPs. Rutile TiO_2_ NPs caused significant changes in cell morphology and function. Confocal microscopy revealed a decrease in cell area and altered cell morphology, with cells appearing elongated and detached from the surface after exposure to rutile TiO_2_ NPs. This alteration in morphology was associated with thinner and less extended actin fibers, affecting cell proliferation and function. Further experiments demonstrated a reduction in cell migration and collagen contraction ability upon exposure to rutile TiO_2_ NPs. Anatase TiO_2_ NPs exhibited even more pronounced adverse effects, causing damage to actin fibers and cell membranes at lower concentrations and shorter incubation times compared to rutile NPs. Anatase NPs led to a dramatic decrease in cell survival and proliferation. Western blot analysis confirmed the severe impact of anatase TiO_2_ NPs on actin. TEM images showed extensive damage to cell cytoplasm, with some nuclei appearing vesiculated, raising concerns about potential direct harm to DNA molecules [213]. A study conducted by Prasad et al. investigated the effects of TiO_2_ NPs on DNA damage response pathways in human dermal fibroblasts. The study focused on understanding how TiO_2_ NPs exposure influences DNA damage response mechanisms, particularly looking at the activation of ATM/Chk2 and ATR/Chk1 pathways. The findings revealed that exposure to TiO_2_ NPs led to increased phosphorylation of H2AX, ATM, and Chk2, indicating activation of the ATM/Chk2 DNA damage response pathway [214].

The literature reviewed suggests that TiO_2_ NPs exhibit cytotoxic effects on skin cells *in vitro*. However, whether TiO_2_ NPs can penetrate the skin and enter the body remains a subject of ongoing research. Some studies suggest TiO_2_ NPs cannot penetrate skin, while others indicate they may under certain conditions. In a study conducted by Adachi et al., the skin of male hairless Wistar Yagi rats was exposed to a water-in-oil emulsion containing 10 wt% TiO_2_ NPs. After 4 hours of exposure, no morphological or immunohistochemical changes were observed in the skin. The results also demonstrated that TiO_2_ NPs were primarily localized in the interfollicular stratum disjunctum and the keratinized layer of the follicular infundibulum, with none detected in viable skin layers [215]. Crosera et al. discovered that after 24 hours of exposure, TiO_2_ NPs are unable to penetrate intact or damaged human skin samples. They detected titanium content only in the epidermal layer, with concentrations of 0.47 ± 0.33 μg/cm^2^ in intact skin samples and 0.53 ± 0.26 μg/cm^2^ in damaged skin samples. Notably, titanium content was found to be below the limit of detection in the dermal layer [216]. A study by Xie et al. involved exposing ^125^I-labeled TiO_2_ NPs to the skin of male Wistar rats and assessing the radioactivity of blood and tissues of the rats after 1 day and 3 days. The results showed that the radioactivity of blood and tissues of rats after exposure to ^125^I-TiO_2_ NPs solution through slightly damaged skin or intact skin was <0.05% dose/g on Day 1 and quickly declined on Day 3. The findings indicated that TiO_2_ NPs could not penetrate through the slightly damaged or intact skin, suggesting their safety when applied and in contact with the skin [217]. In a study conducted by Wu et al., the penetration and potential toxicity of TiO_2_ NPs were investigated through both *in vitro* and *in vivo* experiments. *In vitro* experiments using isolated porcine skin showed that TiO_2_ NPs did not penetrate the stratum corneum after 24 hours of exposure. However, contrasting results were obtained *in vivo* when pig ear skin was topically treated with TiO_2_ NPs for 30 days; here, TiO_2_ NPs were able to penetrate deeper layers of the epidermis. Additionally, hairless mice exposed to TiO_2_ NPs for 60 days exhibited penetration through the skin, reaching different tissues and inducing pathological lesions in various organs, particularly the skin and liver. Notably, TiO_2_ NPs were found to induce oxidative stress, as evidenced by decreased activity of superoxide dismutase and an increased level of malondialdehyde. The results also indicated that hydroxyproline content was reduced, suggesting that prolonged exposure to TiO_2_ NPs can induce skin aging. These findings highlight the potential health risks associated with prolonged dermal exposure to nanoscale TiO_2_ particles [218].

Despite all efforts, there remains a significant amount of uncertainty surrounding the toxicity of TiO_2_ NPs. This uncertainty arises from the complexity of biological systems and the challenge of predicting how TiO_2_ NPs will interact with them. This underscores the importance of conducting further research to comprehensively understand and mitigate the potential hazards associated with these nanomaterials.

## Conclusions

The management of skin wounds remains a clinical challenge, highlighting the need for more effective treatments. In response, a growing body of research has explored the use of nanobiomaterials for wound care. In this context, TiO_2_ NPs have demonstrated promising biological properties—such as antibacterial, antifungal, antioxidant, and anti-inflammatory effects—making them suitable for wound healing applications. Several studies have introduced approaches that leverage a variety of biomaterials and fabrication methods to develop scaffolds and dressings incorporating TiO_2_ NPs, supporting the multifactorial nature of the wound healing process. Despite the promising outcomes, concerns remain regarding the toxicity of TiO_2_ NPs at high concentrations and with prolonged exposure. The complexity of biological systems further complicates predictions about how TiO_2_ NPs interact within these systems. These concerns underscore the need for further investigation to fully elucidate the potential risks and mechanisms of toxicity associated with TiO_2_ NPs.

## Data Availability

No data was used for the research described in the article.
